# Supplementary figures and images for: Bases of antisense lncRNA-associated regulation of gene expression in fission yeast
Source: PLoS Genet. 2018 Jul 5;14(7):e1007465. doi: 10.1371/journal.pgen.1007465 (PMC6049938; doi:10.1371/journal.pgen.1007465)

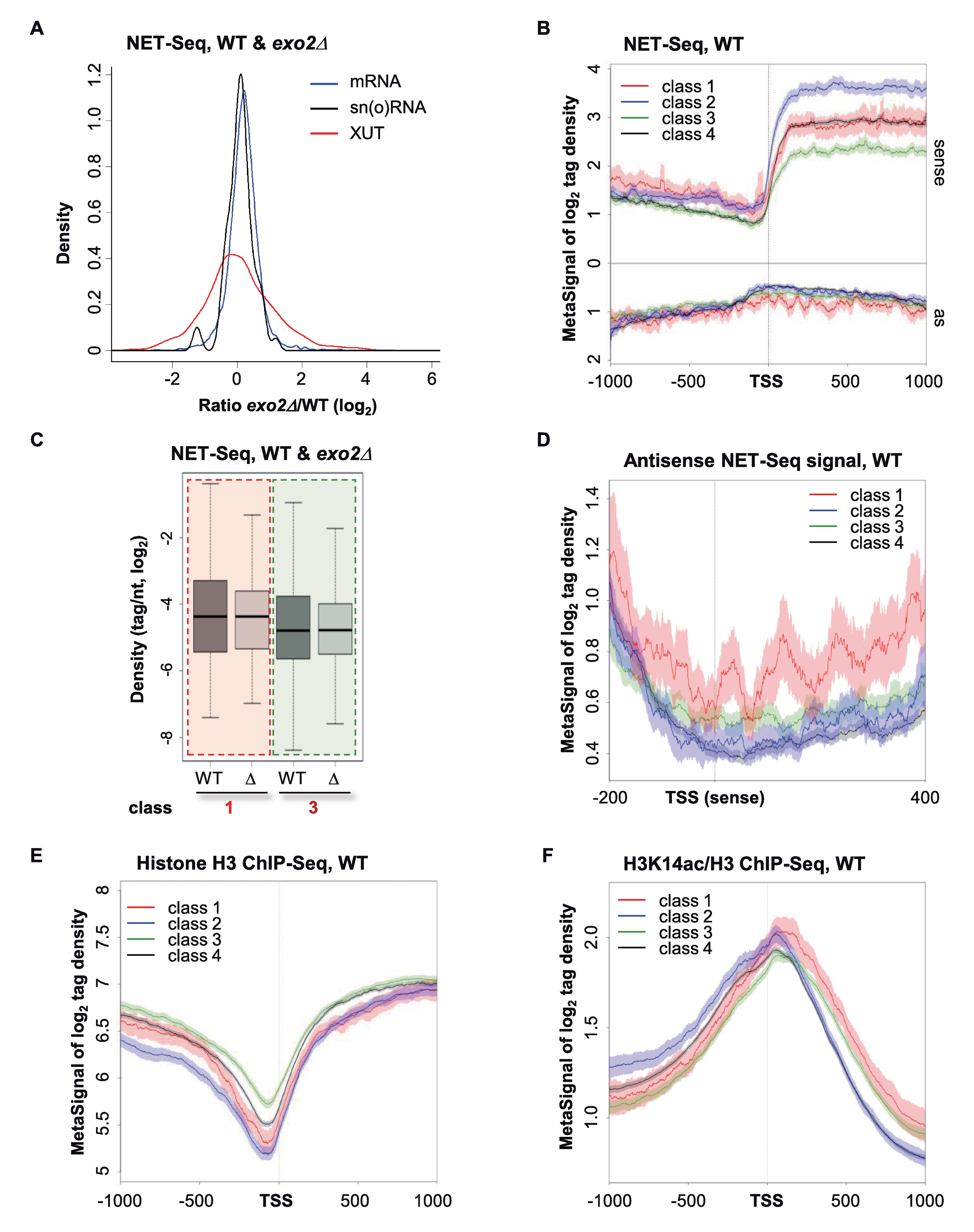

Supplement: S1 Fig — A. Global RNAPII transcription in WT and exo2Δ cells. Density plot of exo2Δ/WT NET-Seq signal ratio for mRNAs (blue), sn(o)RNAs (black) and XUTs (red). B. Metagene view of NET-Seq signals along class 1–4 genes in WT cells. For each class, normalized signal (tag/nt, log2) along mRNA transcription start site (TSS) +/- 1000 nt (sense strand) and the antisense (as) strand were piled up, in a strand-specific manner. Average signal for each strand was plotted for class 1 (red), 2 (blue), 3 (green) and 4 (black). The shading surrounding each line denotes the 95% confidence interval. C. Box-plot of NET-Seq signal (tag/nt, log2) for XUTs antisense to class 1 and class 3 genes in WT and exo2Δ (Δ) cells. D. Metagene view of nascent antisense transcription (NET-Seq) signal around the sense gene TSS of class 1–4 genes, in WT cells. The shading surrounding each line denotes the 95% confidence interval. E. Metagene of H3 levels for class 1–4 genes in WT cells. The analysis was performed using previously published ChIP-Seq data [30]. Metagene representation of signal for class 1 (red), class 2 (blue), class 3 (green) and class 4 (black) was performed as above, in a strand-unspecific manner. The shading surrounding each line denotes the 95% confidence interval. F. Metagene view of H3K14 acetylation for class 1–4 genes in WT cells. ChIP-Seq libraries construction and sequencing were previously described [30]. Metagene representation of signal for each class of genes was performed as above, using ratio of coverage (log2) for H3K14ac and H3. (TIF) [file pgen.1007465.s001.tif]

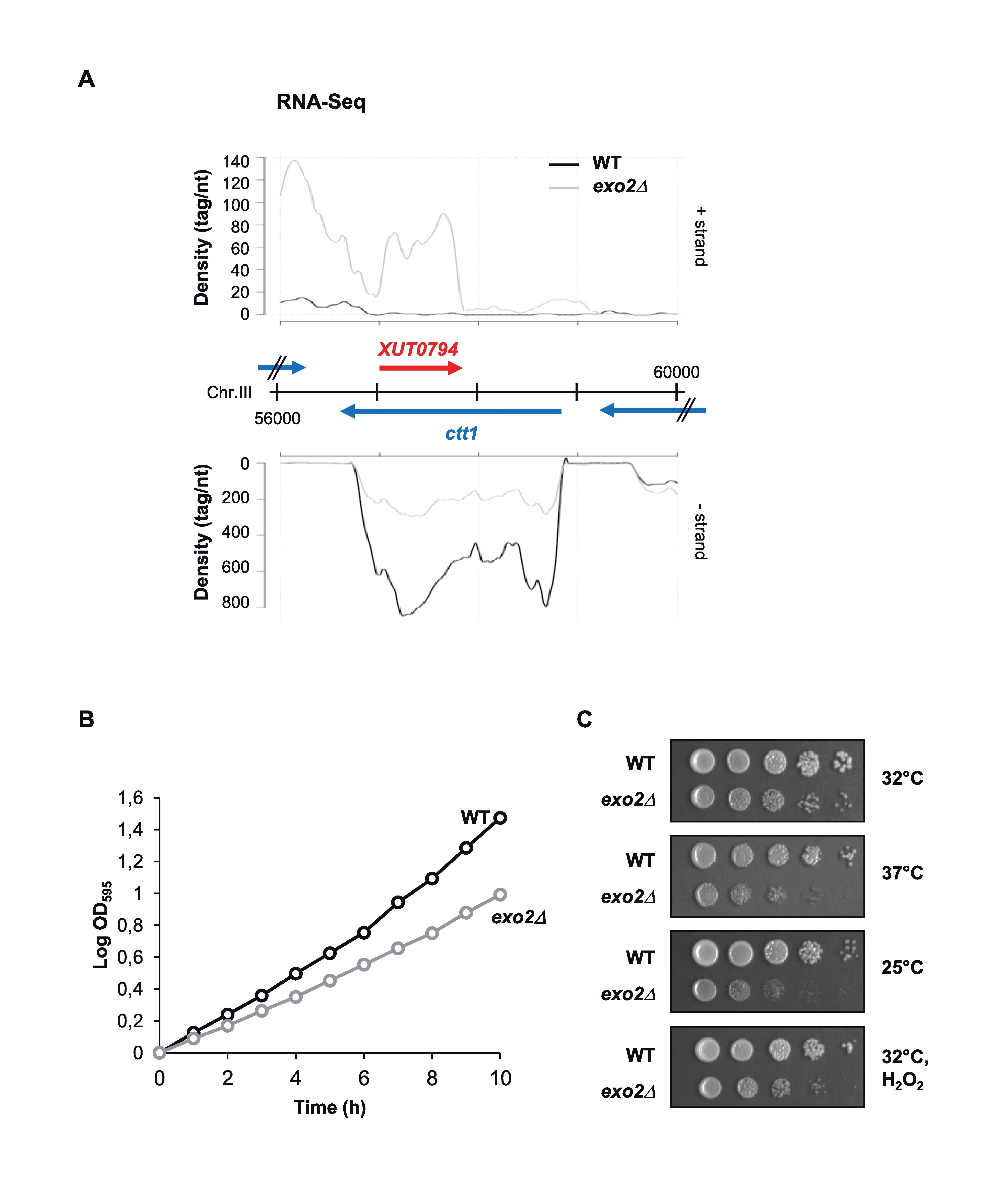

Supplement: S2 Fig — A. Snapshot of total RNA-Seq signals along the ctt1 locus in WT and exo2Δ cells. RNA-Seq data were previously published [30]. Densities (tag/nt) for the + and—strands are visualized in the upper and lower panels, respectively. The signals for the WT and the exo2Δ strains are represented as black and grey lines, respectively. B. Exo2-deficient cells display a slow growth phenotype. YAM2400 (WT) and YAM2402 (exo2Δ) cells were grown in rich (YES) medium, at 32°C. OD595 was measured every hour. OD595 at time 0 was set to 1, for each strain. Data are expressed in a log scale. C. Loss of Exo2 confers sensitivity to hydrogen peroxide. Serial 1:10 dilutions of YAM2400 (WT) and YAM2402 (exo2Δ) cells were dropped on solid rich medium (YES) containing or not 2 mM H2O2. Plates were incubated at the indicated temperature for 3–4 days. (TIF) [file pgen.1007465.s002.tif]

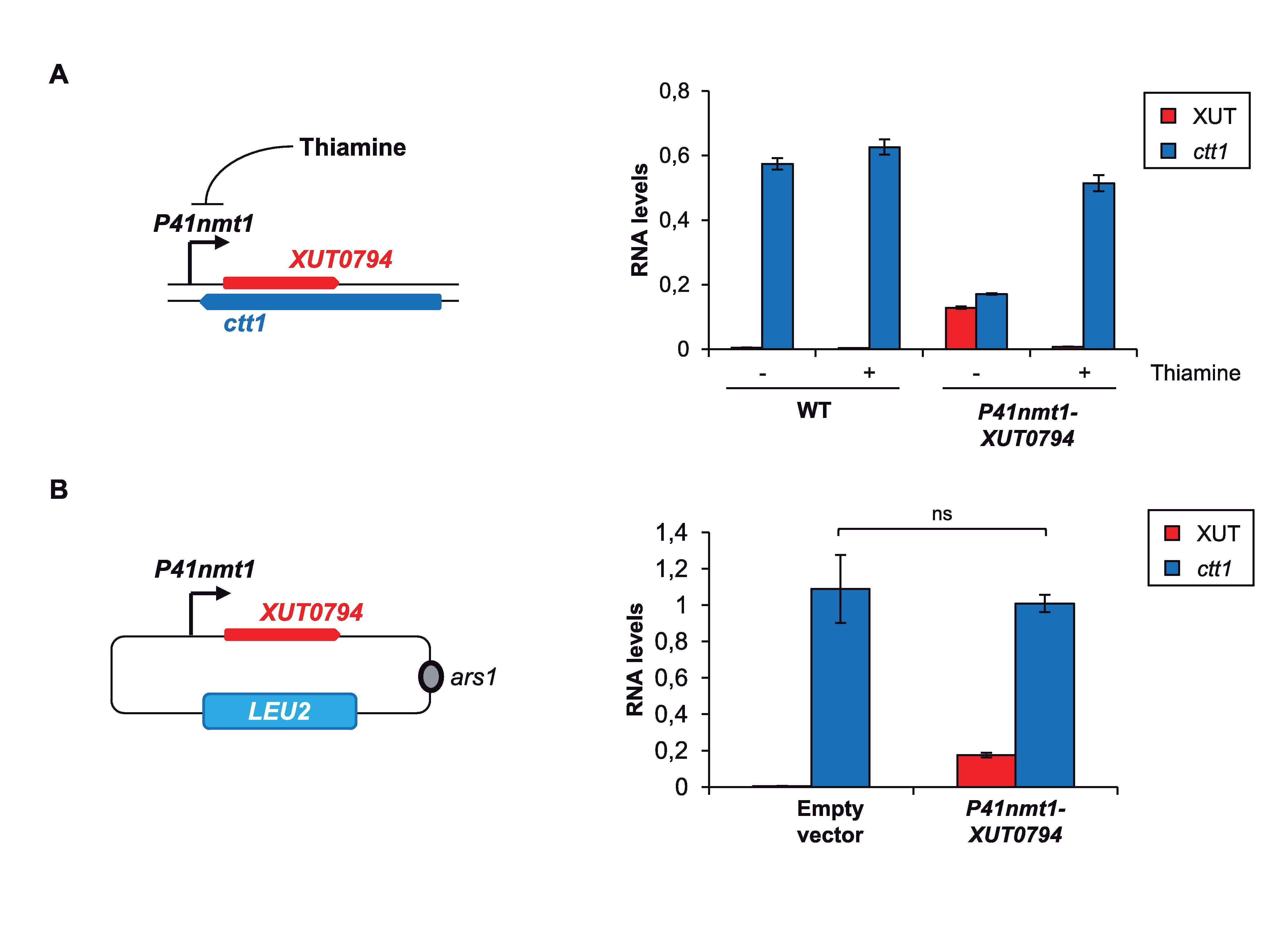

Supplement: S3 Fig — A. Attenuation of ctt1 mRNA upon overexpression of XUT0794 in cis. Strains YAM2400 (WT) and YAM2474 (P41nmt1-XUT0794) were grown for 24 hours to mid-log phase in EMM medium +/- 15 μM thiamine, before addition of H2O2 for 15 min. Levels of XUT0794 (red) and ctt1 mRNA (blue) were quantified from total RNA using strand-specific RT-qPCR and normalized on the level of the U3B snoRNA. Data are presented as mean +/- SEM from three biological replicates. B. Induction of ctt1 mRNA upon XUT0794 overexpression in trans. YAM2475 (empty vector) and YAM2476 (pAM353; P41nmt1-XUT0794) cells were grown for 24 hours to mid-log phase in EMM-L medium, before addition of H2O2 for 15 min. Determination of XUT0794 and ctt1 mRNA levels and data presentation are as above. (TIF) [file pgen.1007465.s003.tif]

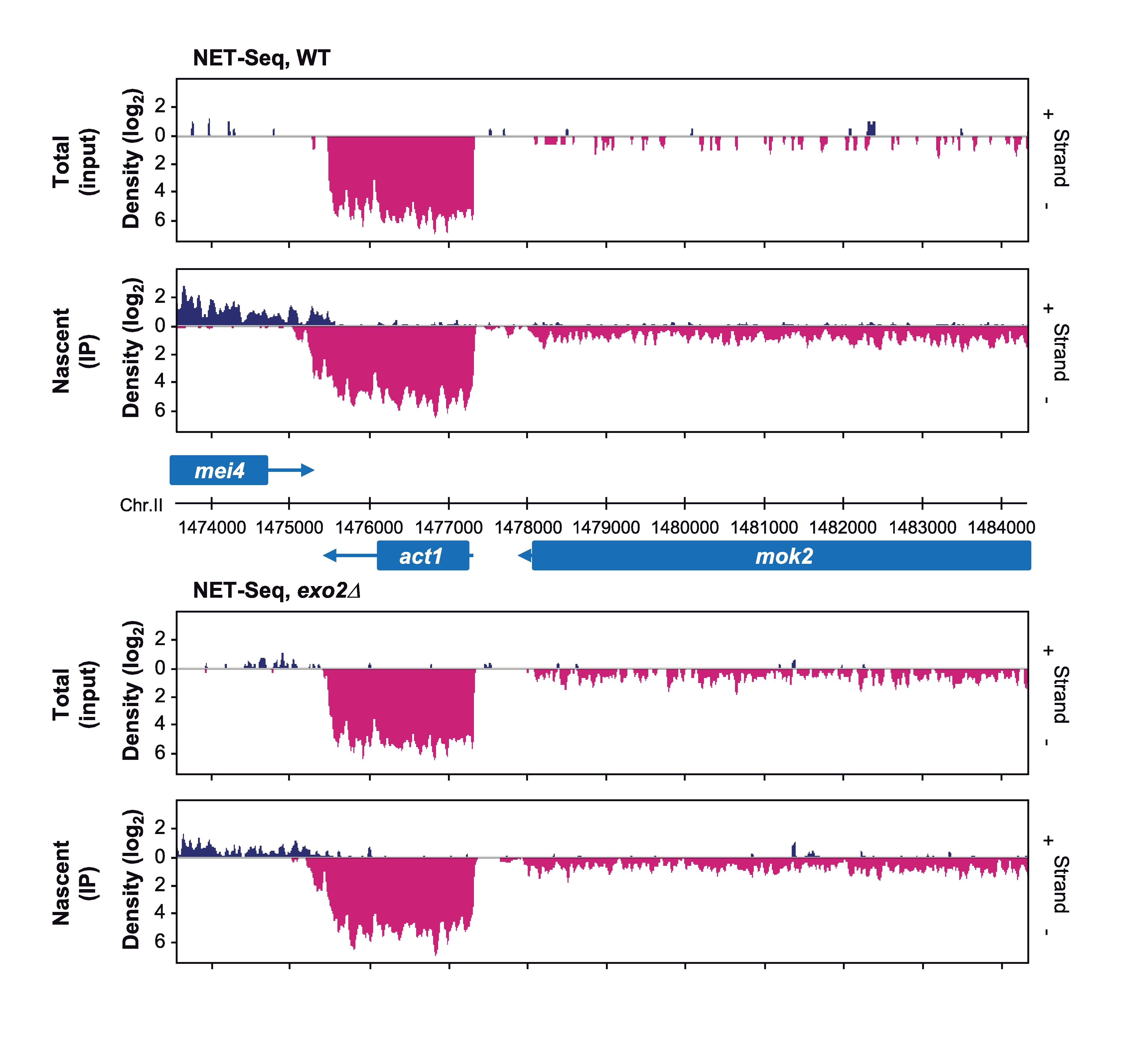

Supplement: S4 Fig — Snapshot of total (input) and nascent (IP) NET-Seq signals along the act1 gene in WT (upper panels) and exo2Δ (lower panels) cells. In each panel, the signal corresponding to the sense (+) and antisense (-) strand is shown in blue and pink, respectively. Blue arrows and boxes represent the mRNAs and coding sequences, respectively. NET-Seq data for the WT strain were previously described [30]. The snapshot was produced using VING [59]. (TIF) [file pgen.1007465.s004.tif]

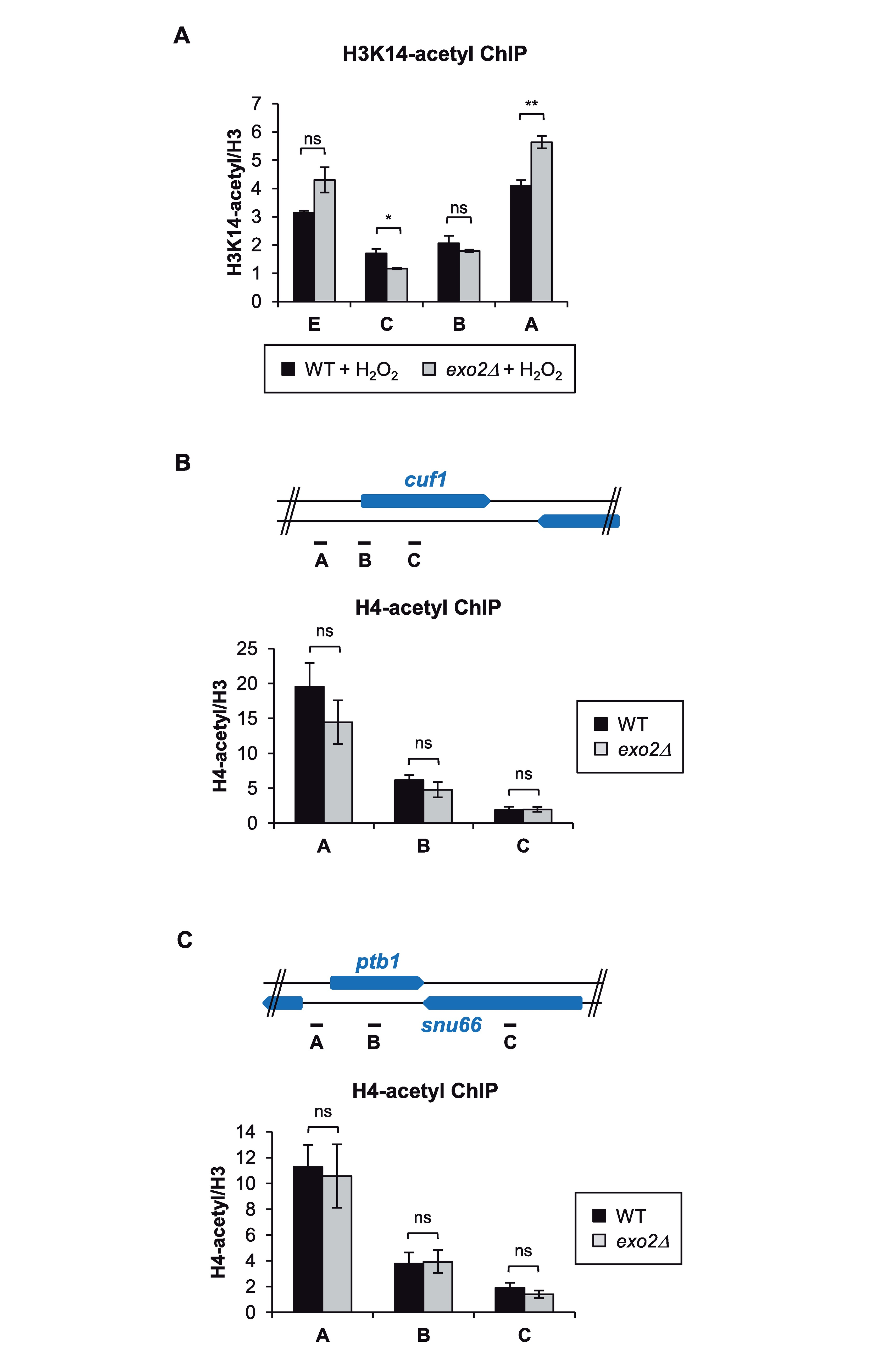

Supplement: S5 Fig — A. ChIP analysis of H3K14 acetylation along ctt1. Culture, cross-linking and chromatin extraction were as described in Fig 4B. For each position, data were first normalized on act1, then on the level of histone H3, immunoprecipitated from the same chromatin. Data are presented as mean +/- SEM, calculated from three biological replicates. *p<0.05; **p<0.01; ns, not significant upon t-test. B-C. ChIP analysis of H4 K5/8/12/16 acetylation along the class 2 genes cuf1 (B) and ptb1 (C) in WT and exo2Δ cells. Cultures were as described in Fig 5D. Cross-linking, chromatin extraction, data analysis and presentation were as above. ns, not significant upon t-test. (TIF) [file pgen.1007465.s005.tif]

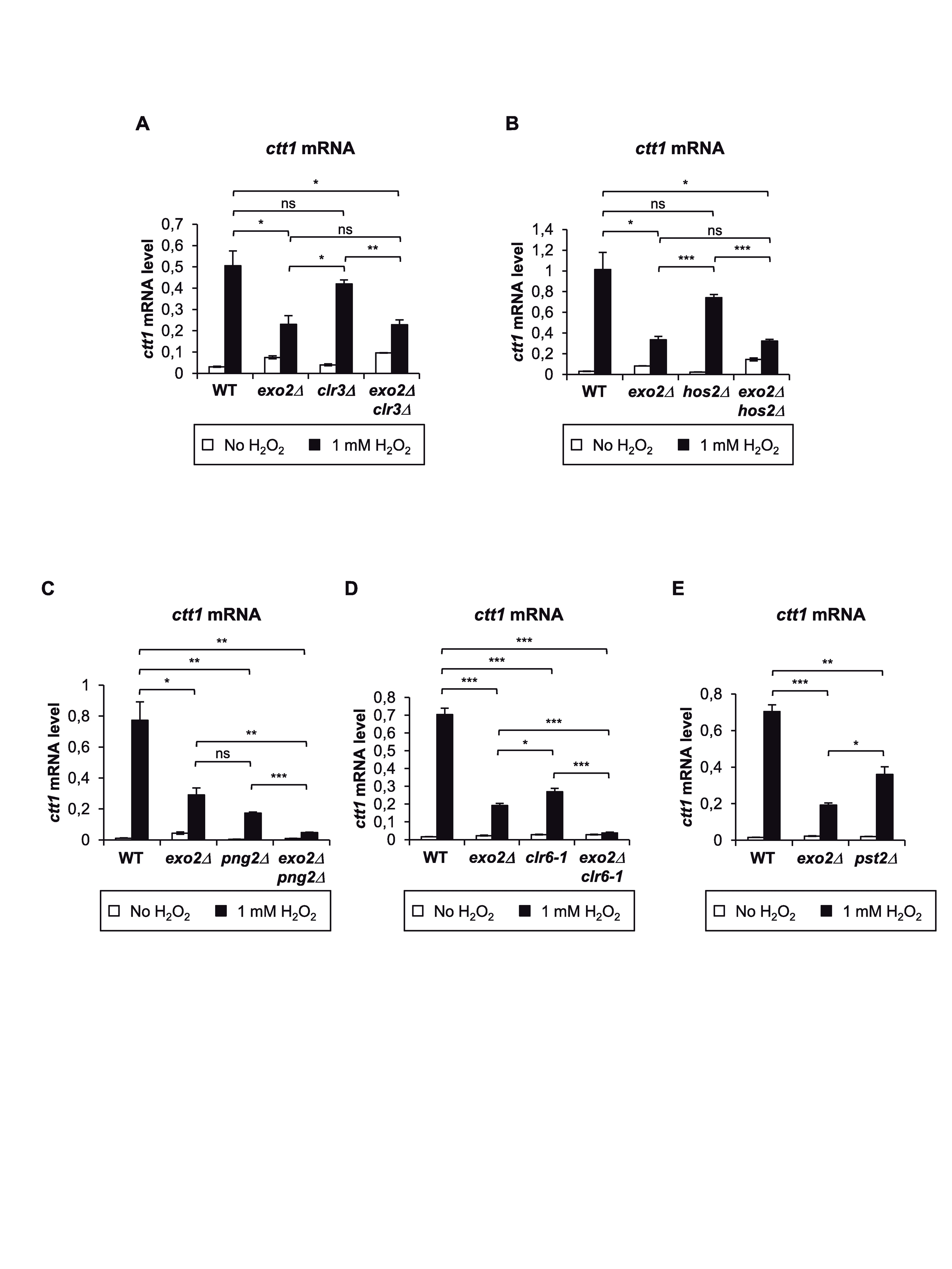

Supplement: S6 Fig — A. Effect of the Clr3 class II HDAC. YAM2400 (WT), YAM2402 (exo2Δ), YAM2407 (clr3Δ) and YAM2444 (exo2Δ clr3Δ) cells were grown in rich medium before (white) or after addition of 1 mM H2O2 for 15 minutes (black). ctt1 mRNA levels were quantified by strand-specific RT-qPCR from total RNA as described in Fig 2C. Data are presented as mean +/- SEM from three biological replicates. *p<0.05; **p<0.01; ns, not significant upon t-test. B. Effect of Hos2 class I HDAC. Same as above using YAM2400 (WT), YAM2402 (exo2Δ), YAM2471 (hos2Δ) and YAM2472 (exo2Δ hos2Δ). *p<0.05; ***p<0.001; ns, not significant upon t-test. C. Effect of the Png2 subunit of the Clr6CI complex. Same as above using YAM2400 (WT), YAM2402 (exo2Δ), YAM2561 (png2Δ) and YAM2562 (exo2Δ png2Δ). *p<0.05; **p<0.01; ***p<0.001; ns, not significant upon t-test. D. Effect of Clr6 class I HDAC. Same as above using YAM2400 (WT), YAM2402 (exo2Δ), YAM2798 (clr6-1) and YAM2814 (clr6-1 exo2Δ). *p<0.05; ***p<0.001 upon t-test. E. Effect of the Pst2 subunit of the Clr6CII complex. Same as above using YAM2400 (WT), YAM2402 (exo2Δ) and YAM2815 (pst2Δ). *p<0.05; **p<0.01; ***p<0.001 upon t-test. (TIF) [file pgen.1007465.s006.tif]

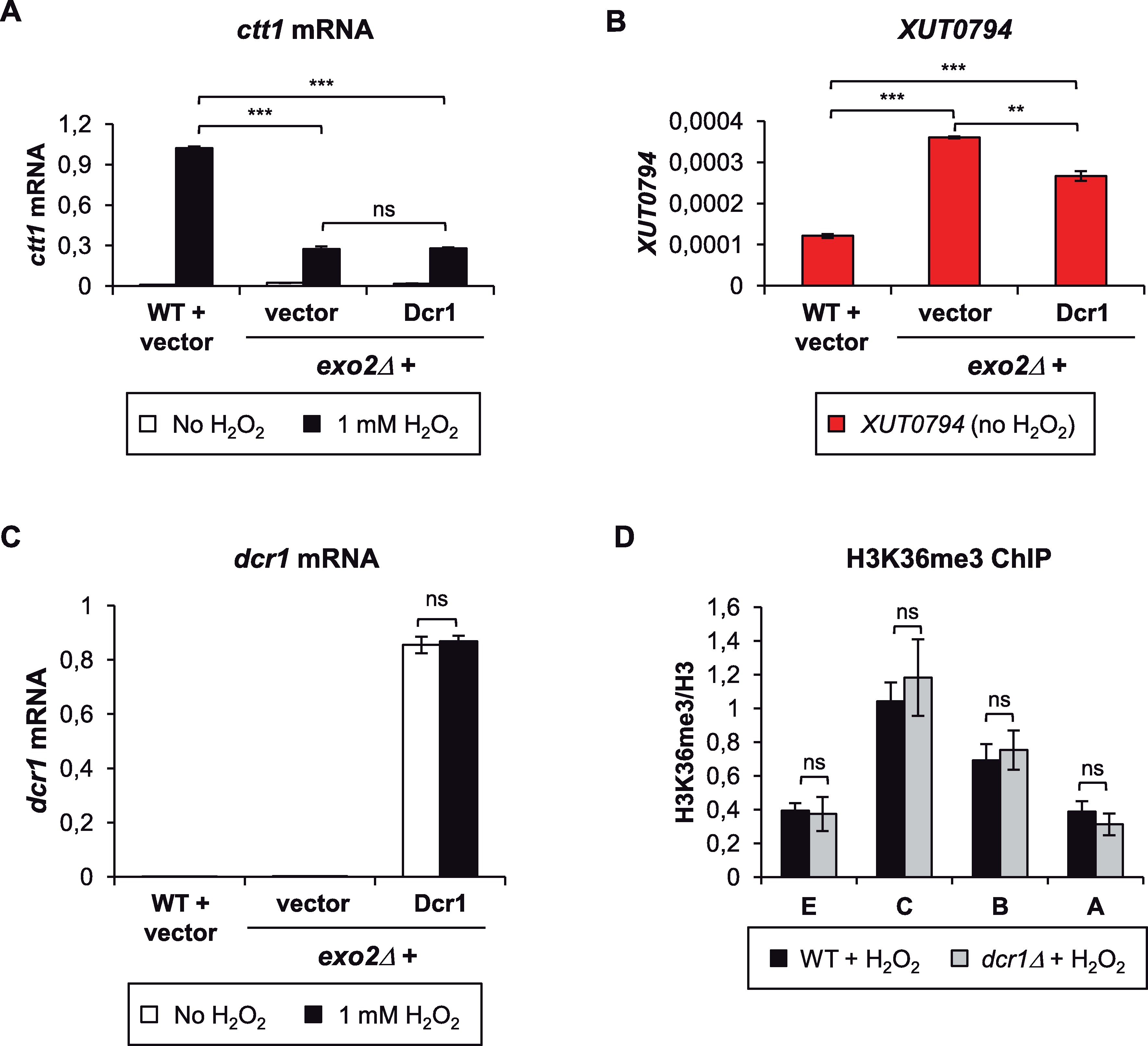

Supplement: S7 Fig — A-C. WT cells with pREP-nmt1/LEU2 empty vector (pDM829, vector), and exo2Δ cells with pREP-nmt1/LEU2 or pREPNFLAG-Dcr1 (pDM914, Dcr1) plasmids [61] were grown to mid-log phase in EMM-Leu medium, before addition of H2O2 for 15 min. ctt1 mRNA (A), XUT0794 (B) and dcr1 mRNA (C) were quantified from total RNA using strand-specific RT-qPCR and normalized on the level of the U3B snoRNA. Average values and SEM were calculated from three biological replicates. **p<0.01; ***p<0.001; ns, not significant upon t-test. D. ChIP analysis of H3K36 trimethylation (H3K36me3) along ctt1 in dcr1Δ cells. Strains YAM2400 (WT) and YAM2406 (dcr1Δ) were grown as in Fig 2B. Cross-linking and chromatin extraction were as described in Fig 4B. Data analysis was performed as described in Fig 5C. Data are presented as mean +/- SEM, calculated from three biological replicates. ns, not significant upon t-test. (TIF) [file pgen.1007465.s007.tif]
