# Supplementary material for: Bases of antisense lncRNA-associated regulation of gene expression in fission yeast
Source: PLoS Genet. 2018 Jul 5;14(7):e1007465. doi: 10.1371/journal.pgen.1007465 (PMC6049938; doi:10.1371/journal.pgen.1007465)
Supplement: S5 Table — (PDF) [file pgen.1007465.s012.pdf]

S5 Table. Yeast strains.

Strain ID	Genotype	Source/Reference
YAM2400	*h⁻*	(30)
YAM2401	*h^+ exo2Δ::kan^R*	This work
YAM2402	*h^- exo2Δ::kan^R*	(30)
YAM2404	*h^- exo2Δ::kan^R dcr1Δ::nat^R*	This work
YAM2406	*h^- dcr1Δ::nat^R*	This work
YAM2407	*h^- clr3Δ::nat^R*	This work
YAM2444	*h^- exo2Δ::kan^R clr3Δ::nat^R*	This work
YAM2471	*h^- hos2Δ::hph*	This work
YAM2472	*h^+ exo2Δ::kan^R hos2Δ::hph*	This work
YAM2474	*h^- nat^R- P41nmt1-XUT0794*	This work
YAM2475	*h^+ ade6-216 ura4-D18 leu1-32 + pAM342/pREP41*	This work
YAM2476	*h^+ ade6-216 ura4-D18 leu1-32 + pAM353*	This work
YAM2492	*h^- rpb3-flag::nat^R*	This work
YAM2507	*h^- exo2Δ::kan^R rpb3-flag::nat^R*	This work
YAM2534	*h^+ ade6-216 ura4-D18 leu1-32 ctt1::ura4*	This work
YAM2561	*h^+ png2Δ::kan^R*	This work
YAM2562	*h^- exo2Δ::kan^R png2Δ::kan^R*	This work
YAM2565	*h^+ ade6-216 ura4-D18 leu1-32 XUT0794-RZ*	This work
YAM2567	*h^+ ade6-216 ura4-D18 leu1-32 exo2Δ::nat^R XUT0794-RZ*	This work
YAM2797	*h^- set2::kan^R*	This work
YAM2798	*h^- clr6-1*	(38)
YAM2814	*h^- clr6-1 exo2Δ::kan^R*	This work
YAM2815	*h^- pst2::kan^R*	This work
YAM2816	*h^- ade6-216 leu1-32 ura4-D18 set2::13Myc-Kan^R*	(60)
YAM2817	*h^+ ura4-XUT0794*	This work
