# Supplementary material for: Bases of antisense lncRNA-associated regulation of gene expression in fission yeast
Source: PLoS Genet. 2018 Jul 5;14(7):e1007465. doi: 10.1371/journal.pgen.1007465 (PMC6049938; doi:10.1371/journal.pgen.1007465)
Supplement: S6 Table — (PDF) [file pgen.1007465.s013.pdf]

S6 Table. **Oligonucleotides.**

**ID**	**Sequence 5'-3'**	**Target**	**Use**
AMO2063	TGGAAAAGCCATCCATGAAACGG	*S. pombe ctt1*	Northern blot
AMO2065	CACCGGTTGTCCCATTTTTA	*S. pombe ctt1*	qPCR (probe B)
AMO2066	CTCGCGATCAAAGTGTTGAA	*S. pombe ctt1*	qPCR (probe B)
AMO2069	CCCTCAGGAGGCTGAAAAAT	*S. pombe ctt1*	RT (XUT) + qPCR (probe C)
AMO2070	TCAAGGTAAAGCGTCCAACC	*S. pombe ctt1*	RT (mRNA) + qPCR (probe C)
AMO2080	CTTTGAGGATGTGCTGATCG	*S. pombe U3B*	qPCR
AMO2081	CATGACACGACCAAAAGGAA	*S. pombe U3B*	Northern-blot, RT & qPCR
AMO2094	TACGTTAGCGATGGTGTTGG	*S. pombe ctt1*	qPCR (probe A)
AMO2095	AGCCTATCGTAGTCGCCAAA	*S. pombe ctt1*	qPCR (probe A)
AMO2098	GTTTGGCCGGTTTATCAATG	*S. pombe ctt1*	qPCR (probe E)
AMO2099	GACACTCGAGTTCTCCCAATG	*S. pombe ctt1*	qPCR (probe E)
AMO2280	TTGCAAACAATTTGCACACAA	*S. pombe chr.I:* 3953119-99	qPCR
AMO2281	TTCTAGTAGAACGCCAGTCTTCAA	*S. pombe chr.I:* 3953119-99	qPCR
AMO2350	CCACTATGTATCCCGGTATTGC	*S. pombe act1*	qPCR
AMO2351	CAATCTTGACCTTCATGGAGCT	*S. pombe act1*	qPCR
AMO2426	AACCCTCAGCTTTGGGTCTT	*S. pombe act1*	qPCR
AMO2427	TTTGCATACGATCGGCAATA	*S. pombe act1*	qPCR
AMO2515	TCGGGCTGTTAAGACTGCTT	*S. pombe dcr1*	qPCR
AMO2516	ATTGCCAGTTCCTCGTCATC	*S. pombe dcr1*	RT + qPCR
AMO2535	GGCGTTCACGTACAGGAGAT	*S. pombe ctt1*	RT (mRNA) + qPCR (probe D)
AMO2536	AGCAACCTAGAGCGTTTTGG	*S. pombe ctt1*	qPCR (probe D)
AMO2820	CCGTCCCTATTAAACGCAGT	*S. pombe SPAPB24D3.07c*	qPCR (probe A)
AMO2821	CCAACAAGGTCCAAACGAAT	*S. pombe SPAPB24D3.07c*	qPCR (probe A)
AMO2822	ATTCGTTTGGACCTTGTTGG	*S. pombe SPAPB24D3.07c*	qPCR (probe B)
AMO2823	GAAAGAGGGCCACTGGTGTA	*S. pombe SPAPB24D3.07c*	qPCR (probe B)
AMO2824	CTGACGCTTTGGTTGGTACA	*S. pombe SPAPB24D3.07c*	qPCR (probe C)
AMO2825	TGCTGATCTCGTTGAGGCTA	*S. pombe SPAPB24D3.07c*	qPCR (probe C)
AMO2828	TAAGCCAACACCCAGTTTCC	*S. pombe cuf1*	qPCR (probe A)
AMO2829	GCCCAAAAAGCAGTTTTCAA	*S. pombe cuf1*	qPCR (probe A)
AMO2830	CTCCCATTGAGCTTCCATTT	*S. pombe cuf1*	qPCR (probe B)
AMO2831	CACTTCATGCATGCCATTTT	*S. pombe cuf1*	qPCR (probe B)
AMO2832	AAACGCATTGTCCAAAAAGG	*S. pombe cuf1*	qPCR (probe C)
AMO2833	CGATAGGATTCCGAATGTGG	*S. pombe cuf1*	qPCR (probe C)
AMO2834	CCACCACATTCGTAAATGGA	*S. pombe ptb1*	qPCR (probe A)
AMO2835	ACGGTTCGTTATGCAATGGT	*S. pombe ptb1*	qPCR (probe A)
AMO2836	TGGAGCTGAGTCACATGGAG	*S. pombe ptb1*	qPCR (probe B)
AMO2837	TTCAGGGCGACCATTTAATC	*S. pombe ptb1*	qPCR (probe B)
AMO2838	CATTCGGTAGCACGAAATCA	*S. pombe ptb1*	qPCR (probe C)
AMO2839	CTATCACTGGCAGCGAACAA	*S. pombe ptb1*	qPCR (probe C)
